# The molecular mechanisms of chemoresistance in cancers

**DOI:** 10.18632/oncotarget.19048

**Published:** 2017-07-06

**Authors:** Hua-Chuan Zheng

**Affiliations:** ^1^ Department of Experimental Oncology and Animal Center, Shengjing Hospital of China Medical University, Shenyang 110004, China

**Keywords:** cancer, chemoresistance, molecular mechanisms, chemotherapy

## Abstract

Overcoming intrinsic and acquired drug resistance is a major challenge in treating cancer patients because chemoresistance causes recurrence, cancer dissemination and death. This review summarizes numerous molecular aspects of multi-resistance, including transporter pumps, oncogenes (EGFR, PI3K/Akt, Erk and NF-κB), tumor suppressor gene (p53), mitochondrial alteration, DNA repair, autophagy, epithelial-mesenchymal transition (EMT), cancer stemness, and exosome. The chemoresistance-related proteins are localized to extracellular ligand, membrane receptor, cytosolic signal messenger, and nuclear transcription factors for various events, including proliferation, apoptosis, EMT, autophagy and exosome. Their cross-talk frequently appears, such as the regulatory effects of EGFR-Akt-NF-κB signal pathway on the transcription of Bcl-2, Bcl-xL and survivin or EMT-related stemness. It is essential for the realization of the target, individualized and combine therapy to clarify these molecular mechanisms, explore the therapy target, screen chemosensitive population, and determine the efficacy of chemoreagents by cell culture and orthotopic model.

## INTRODUCTION

Chemoresistance causes disease relapse and metastasis, challenges the improvement of clinical outcome for the cancer patients, and remains the main obstacle to cancer therapy. Therefore, it is very important to understand its molecular mechanisms, and find out novel therapeutic approaches for cancer therapy. According to our knowledge, the molecular mechanisms of chemoresistance include transporter pumps, oncogenes, tumor suppressor gene, mitochondrial alteration, DNA repair, autophagy, epithelial-mesenchymal transition (EMT), cancer stemness, and exosome [1, 2]. There appear many cross talks between these aspects as shown in the context. For example, the encoding proteins of oncogenes (EGFR-Akt-NF-κB) might modulate the apoptosis-related genes expression, and contribute to EMT, cell stemness and autophagy. Autophagic cells are characterized by anti-apoptosis during chemoresistance. The exosome might contain many proteins, which are related to anti-apoptosis and transporter pumps. Additionally, cells will appear stem-like once the onset of EMT when EGF binds to and activates EGFR. Therefore, we speculate that the inhibition of weak- and no-talk signal pathways might synergistically ameliorate the side effects of drugs or therapeutics, and strengthen the anti-tumors efficacy of these treatments.

### Transporter

ABC proteins are members of transport system superfamily and responsible for the translocation of various substrates (e.g. ions, amino acids, peptides, lipids, sugars, and xenobiotic) across cellular membranes using ATPase transporter or channel protein. The structural architecture of ABC transporters minimally contains 2 transmembrane domains and 2 nucleotide-binding domains. The former recognizes and transports a various kinds of substrates across the cellular membrane via conformational changes. On the other hand, the latter has ATP binding site. The proteins include P-glycoprotein (P-gp), ABCG2 and MVP [3].

P-glycoprotein (P-gp, ABCB1 or MDR1) function as an ATP-dependent efflux pump in intestinal epithelium, liver cells, renal proximal tubule, and capillary endothelial cells. It transports a broad range of substrates, including colchicine, tacrolimus, quinidine, etoposide, doxorubicin, vinblastine, lipids, steroids, bilirubin, digoxin, and dexamethasone. Additionally, it might detoxificate cytotoxic drugs in cancer cells via efflux and GSH [2]. P-gp overexpression has been observed in different kinds of hematological and solid tumors, such as leukemia, neuroblastomas, ovarian and breast cancers, demonstrating its contribution to chemoresistance [4, 5].

Breast cancer resistance protein (BCRP/ABCG2, also named as ABCP or MXR1) is a member of ABC superfamily [6]. Its overexpression may been observed in normal tissues for the efflux of cytotoxic drugs (mitoxantrone, daunorubicin, doxorubicin, topotecan and etc.), including the placenta, intestine, liver, blood-testis or -brain barrier, hematopoietic progenitor and other stem cells, as well as chemoresistant cancer cells [2]. ABCG2 was overexpressed in the mitoxantrone (MX)-resistant MCF-7/MX in comparison to the normal parental cells. Estrogen up-regulated the tolerance of MCF-7 cancer cells to MX by inducing ABCG2 expression, but not after the inhibition of estrogen receptor α (ERα). These findings indicated that estrogen induced ABCG2 expression through ERα, and ABCG2 overexpression made MCF-7 more tolerant to MX [7].

MVP (major vault protein) was also known as LRP (lung resistance-related protein), which was firstly discovered as a new 110 kD drug transporter in doxorubicin-resistant lung cancer cells [8]. It is also localized to nuclear pore complexes and interacts with ERα, PTEN and PARP. Therefore, MVP may mediate chemoresistance by modulating the nucleocytoplasmic transport, such as hormones, ribosomes, mRNA and drug. Its overexpression was demonstrated in non-small cell lung carcinoma (NSCLC), B-cell lymphoma and gliomas [2]. It is thought to transport cytotoxic DNA-targeting drugs and mediate primary chemoresistance. MVP might be considered as an independent marker to predict chemoresistance and clinical outcome in the patients with acute leukemia or ovarian cancer [9, 10]. MVP-specific antisense oligonucleotides and anti- LRP monoclonal antibody were found to increase cellular ciplastin level and induce its chemosensitivity of ovarian cancer cells [11].

### Oncogenes

### Growth factor receptor

**Figure 1:**
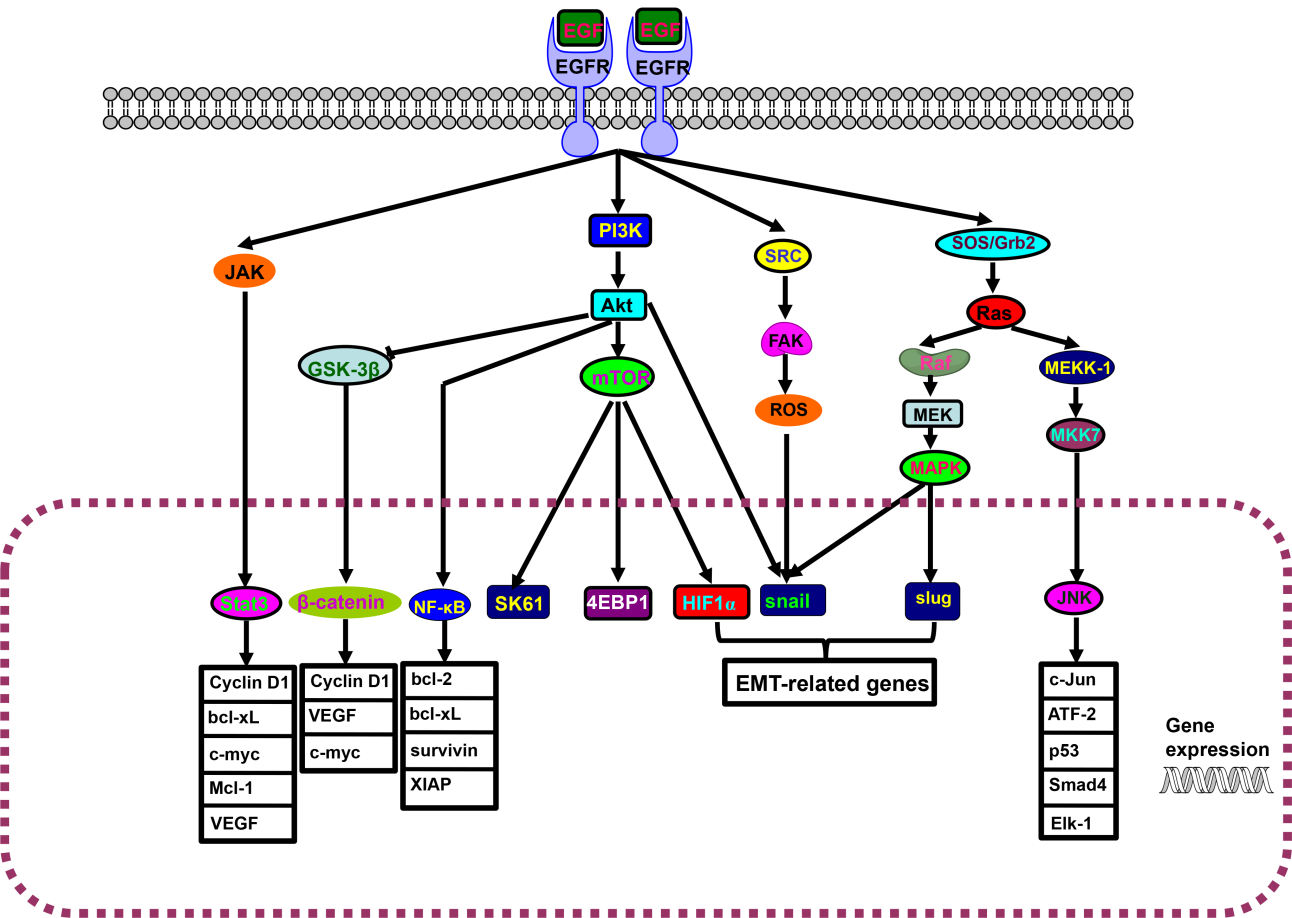
EGFR-Akt-NF-κB pathway The interaction of epidermal growth factor receptor (EGFR) and its ligand induces the dimerization, activation of intrinsic kinase activity and subsequent autophosphorylation of EGFR Tyr 1092, Tyr 1172, Tyr 1197, Tyr 1110, Tyr 1016. JAK/stat3, PI3K/Akt/mTOR and src/FAK/ROS, SOS/Grb2/Ras pathways are triggered to result in the cytonuclear translocation of Stat3, β-catenin, NF-κB, SK61, 4EBP1, HIF1α, snail, slug and JNK for the transcriptional regulation of downstream genes.

Epidermal growth factor receptor (EGFR) can activate JAK/stat3, PI3K/Akt/mTOR and src/FAK /ROS and SOS/Grb2/Ras pathways, and is involved in differentiation, proliferation, survival and transformation (Figure 1). EGFR overexpression can activate NF-κB and STAT3, which leads to chemoresistance and poor outcome. Its mutant is still active and confers the chemoresistance of glioma and lung cancer. However, gefitinib particularly targets mutatant EGFR in lung cancer, and suppresses EGF-triggered and HER3-mediated Akt activation in chemoresistant cells [12]. Recently, Kuroda et al. [13] reported that cisplatin resistance was associated with heme oxygenase (HO)-1 in lung cancer cells through EGFR-mediated PI3K/Akt and NF-κB pathways, which is restored by EGFR-selective tyrosine kinase or Akt inhibitor. The exposure to anti-EGFR monoclonal antibody C225 reduced EGFR expression and the phosphorylation of its downstream Akt and MAPK for the reversal of cellular radioresistance [14]. Tang et al. [15] found that continuous treatment of NSCLC cells with wild-type EGFR to EGFR tyrosine kinase inhibitor induced the chemoresistance to cisplatin, paclitaxel, gemcitabine and pemetrexed by activating STAT3. Further study showed that hippo coactivator YAP1 mediated EGFR overexpression and conferred the resistance to 5-fluorouracil (5-FU) and docetaxcel via an intact TEAD-binding site in EGFR promoter [16]. CD133 overexpression or E3 ubiquitin ligase CBL knockdown conferred the chemoresistance by stabilizing EGFR-Akt signaling or EGFR activation respectively [17, 18]. miR-20b reduced 5-FU resistance for apoptotic induction by inhibiting ADAM9/EGFR pathway in colon cancer cells [19], whereas miRNA-34c-5p inhibited amphiregulin-induced chemoresistance to docetaxel and carboplatin in ovarian cancer cells via the downregulation of AREG-EGFR pathway [20].

### PI3K/Akt

Akt is a serine/threonine-specific protein kinase and involved in apoptosis, proliferation, transcription and migration. Akt phosphorylates BAD protein on ser136 for the disassembly from Bcl-2/Bcl-X partner and suppresses apoptosis-inducing function of BAD. Akt could also activate NF-κB and up-regulate the transcription of pro-survival genes. Akt is also phosphorylated at T308 and S473 and subsequently ubiquitinated partly by E3 ligase NEDD4 for proteasomal degradation. Akt1 overexpression results in drug resistance of NSCLC cells to chemotherapeutic agents. Akt-overexpressing cells displayed the hyperexpression of the antiapoptotic Bcl-xL and delayed the activation of p53 signaling pathway upon the exposure to cisplatin or MX [2, 9]. Cisplatin-induced cytotoxicity caused the phosphorylation of BAD at ser-112 via Erk and BAD at ser-136 via PI3K/Akt, whose block sensitized ovarian cancer cells to cisplatin [21]. Akt1 overexpression and Akt2 gene amplification caused ovarian cancer cells more highly resistant to paclitaxel [22]. Yu et al. [23] demonstrated that PI3K/Akt pathway could be inactivated by doxorubicin and etoposide, and wortmannin could increase the sensitivity of gastric cancer cells to chemotherapy. Activation of endoplasmic reticulum stress was found to increase chemosensitivity of small cell lung cancer cells by suppressing PI3K/Akt/mTOR signaling pathway [24]. You et al. [25] found that trefoil factor 3 enhanced the chemoresistance to doxorubicin in hepatocellular carcinoma (HCC) cells via Akt activation. Luo et al. [26] found that FKBP51 could promote PHLPP-Akt interaction and following Akt dephosphorylation at ser473, so that USP49 enhanced cellular response to gemcitabine through FKBP51-Akt signaling. LncARSR promoted doxorubicin resistance in HCC cells via PTEN-PI3K/Akt pathway [27], and miRNA-130b targeted PTEN to mediate chemoresistance to adriamycin in breast cancer cells via PI3K/Akt pathway [28].

### Erk

Erk1/2 is a widely expressed protein kinase to regulate meiosis, mitosis, and postmitotic function under the stimulation of growth factors, cytokines, virus infection, and ligands for G protein-coupled receptors. After G protein Ras is typically activated by growth factors through receptor tyrosine kinases and GRB2/SOS, Ras can trigger the activation of Raf and following MEK by phosphorylating Thr and Ser. MEK is responsible for the phosphorylation and activation of Erk1/2. After that, Erks are known to activate many transcription factors, such as ELK1, and downstream protein kinases, which are closely linked to cell proliferation, apoptosis and drug resistance. K-ras mutation was reported to result in primary chemoresistance of lung cancer to gefitinib, erlotinib or sunitinib [29]. N-Ras might enhance fibronectin adhesion and chemoresistance of myeloma cells [30]. H-Ras mediated the resistance of ovarian cancer cells against cisplatin by Raf-1/Erk signaling pathway [31]. Reportedly, src-mediated phosphorylation of Caveolin-1 at Y14 was essential for EGFR activation or its interaction with β1 integrin promoted Fyn-dependent Src homology and Erk1/2 phosphorylation, which was closely linked to chemoresistance [32]. Cai et al. [33] found that Erk-mediated phosphorylation of Drp1 at residue S616 contributed to the mitochondrial fusion, a low mitochondrial ROS level, a high proglycolytic shift and drug resistance of T-cell acute lymphoblastic leukemia cells, treated with mesenchymal stem cells. Dong et al. [34] found that Derlin-1 overexpression mediated chemoresistance of bladder cancer through PI3K/Akt and Erk/MMP signaling. Zhang et al. [35] demonstrated that miR-939 contributed to chemosensitivity by inhibiting SLC34A2/ Raf/MEK/Erk pathway.

### NF-κB

NF-κB was firstly discovered by Dr. Ranjan Sen via its interaction with an 11-base pair sequence in the immunoglobulin light-chain enhancer in B cells. NF-κBs have a Rel homology domain in their N-terminus and a transactivation domain in their C-terminus [36]. In unstimulated cells, NF-κB dimers are sequestered in the cytoplasm by IκBs as an inactive form. The activation of NF-κB is initiated by phosphorylation, immediate polyubiquitination and proteasomal degradation of IκB protein [37]. The inducers of NF-κB activity are highly variable, including ROS, TNFα, IL-1β, LPS, isoproterenol, cocaine, ionizing radiation, viruses, and chemoreagents [38]. Active NF-κB enters the nucleus and up-regulates the transcription of Bcl-2, Bcl-xL, XIAP, survivin and Akt (Figure 1). Sun et al. [39] found that cisplatin-resistant bladder cancer cells showed more aggressiveness, rapid tumorigenesis, drug resistance and EMT via NF-κB activation. Körber et al. [40] demonstrated that chemoresistance to 5-FU in this colonic cancer cells was strongly dependent on NF-κB activation. Li et al. [41] found that DNA-PKCs downregulation suppressed P-gp expression for cisplatin chemosensitivity by weakening Akt/NF-κB pathway in osteosarcoma cells with CD133 positive. Aspirin or dehydroxymethylepoxyquinomicin could suppress acquired chemoresistance by disrupting NF-κB-IL6 or NF-κB-ABC transporter axis respectively [42, 43].

### Tumor suppressor gene

### p53

p53 towards cisplatin-induced apoptosis has been confirmed in testicular cancer cells by down-regulating the expression of anti-apoptotic genes and up-regulating the expression of pro-apoptotic genes. Cisplatin treatment increases p53 expression, which transcriptionally targets MDM2, p21Waf1/Cip1 and membrane Fas. Additionally, p53 significantly down-regulates survivin in lung cancer cells by binding to the promoter of survivin for transcriptional suppression, which causes Caspase-3 activation and a decline in cell proliferation with response to adriamycin [9]. In cisplatin-sensitive testicular cancer cell line and its acquired cisplatin-resistant sublines, p53 knockdown decreased cisplatin-induced apoptosis and membrane Fas expression, but versa for intrinsically resistant testicular cancer cell lines [44]. Reportedly, mutant p53 induced drug resistance via miR-223 down-regulation in breast and colon cancer cells by the transcription control of Zeb-1 on miR-223 promoter because miR-223 targets microtubule-modulated stathmin-1 for chemoresistance [45]. Except siRNA transfection, MageA2 protein was shown to suppress p53 transactivation through HDAC3 and MageA2/p53 complex, which was linked to etoposide resistance of melanoma cells [46]. Chen et al. [47] found that β2- adrenergic receptor activation induced chemoresistance by acetylating p53 via Sirt1 in cervical cancer cells. Zhang et al. [48] reported the aggregating p53 mutant Arg282Trp might mediate cisplatin resistance by binding to the promoter of ERP29 and up-regulating its expression. Yao et al. [49] suggested that 53BP1 loss induced chemoresistance of colorectal cancer cells to 5-FU by inhibiting the ATM-CHK2-P53 pathway. Lakshmanan et al. [50] found that MUC16 regulated TSPYL5 through the JAK2/STAT3/GR axis for lung cancer cell chemoresistance to cisplatin and gemcitabine by p53 down-regulation. In contrast, WP1130 attenuated cisplatin resistance via USP9X-p53 ubiquitination-mediated degradation pathway [51].

### Mitochondrial alteration

Mitochondrion is a center of cellular energy powerhouse and represents key intracellular signaling hub of cancer progression, including metabolic reprogramming, acquisition of metastatic capability, and response to chemotherapeutic drugs. The alteration in the functional proteins and morphological dynamics, and genomic DNA would influence mitochondrial events [52].

B-cell lymphoma 2 (Bcl-2) proteins decreases the mitochondrial apoptosis by stabilizing mitochondrial permeability. The pro-apoptotic proteins (Bax, Bak and Bcl-xS) or anti-apoptotic proteins (Bcl-2, Bcl-xL and Mcl-1) induce or inhibit the release of cytochrome c from mitochondrion into the cytosol for the activation of Caspase-9 and -3, leading to apoptosis. Reportedly, Bcl-2 could protect normal cells from toxicity, promote cell survival and cell arrest by up-regulating p27 and p130 expression [53]. Bcl-2 and Bcl-xL enhanced the drug resistance of mesothelioma and laryngeal cancer cells because their down-regulation and Bcl-2 inhibitor (HA14-1) increased the cytotoxic effects of cisplatin, gemcitabine, or the combined treatment with 4625 and cisplatin [9]. Survivin protein belongs to the inhibitor of apoptosis (IAP) family, and can inhibit Caspase activation for negative regulation of apoptosis. Survivin hyperexpression can be employed to predict the chemotherapeutic response of the patients with multiple myeloma, lymphoma, bladder, breast, or ovarian cancer [9]. Survivin expression was positively linked to the extent of cisplatin-resistance in prostate cancer cells, and survivin inhibitor (gambogic acid) might reverse the chemoresistance of gastric cancer cells to docetaxel [9, 54].

The majority of cancer cells harbors somatic mutations in mitochondrial genome (mtDNA), finally to result in mitochondrial dysfunction. Many mutations in mitochondrial genes of cancer cells can't shut down the mitochondrial energy metabolism because some cancer cells may completely depend on more glycolysis and some on more oxidation. Gabrielson et al. [55] found that expression of PGC1α (proliferator-activated receptor gamma co-activator-1α) and TFAM (mitochondrial transcription factor A) might be employed as putative markers of chemoresistance in ovarian cancer. Yao et al. [56] found that PGC-1β mediated adaptive chemoresistance of lung cancer cells to cisplatin associated with mtDNA mutations.

Cardenas et al. [57] found that adipocyte microenvironment promoted Bcl-xL expression and conferred drug resistance of ovarian carcinoma cells. Cisplatin induced the diffusion of mitochondrial proteins (e.g. cytochrome c) through mitochondrial apoptosis-induced channel into the cytosol to initiate the intrinsic apoptosis of ovarian carcinoma cells, which required the mitochondrial translocation of Bax to suppress Bcl-2 protein [58]. Zhou et al. [59] found a low ATP synthesis, a high glycolysis, and a high level of intracellular ATP in chemoreisitant colon cancer cells, which was reversed by an inhibitor of glycolysis (3-bromopyruvate).

Mitochondrial dynamics is characterized by fission and fusion, which allow cellular adaptation to specific metabolic/stem states. During genomic DNA replication, mitochondria are always hyperfused and produce more ATP, but mitochondrial fission is helpful to segregate mitochondrial DNA and eliminate impaired organelles. Mitochondrial fusion with efficient ATP production and transport was more frequently observed in chemoresistant than chemosensitive proportions of gynecological cancer cells. Further study indicated that Drp1 mediated mitochondrial fission, but Mfn 1 and 2, and Opa1 did fusion [60]. Reportedly, both piceatannol and piperlongumine sensitized gynecologic cancer cells to cispltain by inducing Drp1 dephosphorylation at serine 637 by accelerating fission and inducing apoptosis, which was weakened by mdivi (mitochondrial division inhibitor)-1.64. In contrast, Drp 1 down- regulation or depletion sensitized cisplatin-resistant ovarian and lung cancer cells, or inhibited proliferative and autophagic levels of HeLa cells. Drp1 hyperexpression might be employed to predict the relapse and chemoresistance to cisplatin of lung cancer, and linked to aggressiveness of thyroid cancers with fragmented appearances of mitochondria [61]. Oliva et al. [62] demonstrated that temozolomide chemoresistance acquisition was closely correlated with a high mitochondrial coupling and a low ROS production in glioma cells.

### DNA repair

DNA repair is a biological event that cell identifies and corrects the damage to the DNA molecules, induced by endogenous ROS, ultraviolet radiation, x- and gamma rays, plant toxins, mutagenic chemicals, and chemotherapeutic agents. There are two types of DNA repair: nucleotide excision repair and base excision repair, which can confer the drug resistance to DNA-targeting chemodrugs.

ERCC1 forms the ERCC1-XPF enzyme complex that participates in DNA repair and recombination by nucleotide excision repair pathway. ERCC1 overexpression has been negatively linked to the clinical outcome of the patients receiving platinum-based chemotherapy. Li et al. [63] demonstrated that cisplatin regulated the MAPK kinase pathway to induce ERCC1 overexpression and increase melanoma hemoresistance. Zhao et al. [64] found that miR-770-5p inhibited cisplatin chemoresistance in ovarian cancer by targeting ERCC2. McNeil et al. [65] reported that ERCC1- XPF could repair cisplatin-induced DNA lesions in melanoma cells by nucleotide excision repair and interstrand crosslink repair pathways.

Nagel et al. [66] showed that O(6)-methylguanine DNA methyltransferase (MGMT) contributed to acquired temozolomide resistance in orthotopic mouse model of glioblastoma multiforme. Wickström et al. [67] found that MGMT was overexpressed for the development of cancer chemoresistance via Wnt pathway, whose inhibition downregulated MGMT expression and restored the chemosensitivity to DNA-alkylating drugs. As a double-strand break repair protein, MRE11 hypoexpression was positively associated with good response to chemotherapy and surgical resection after down-staging by chemotherapy. Furthermore, the expression of MRE11 and RAD50 (DNA repair protein Rad50) was independent predictors of surgical resection after chemotherapy [68].

The insufficiencies in DNA damage repair were involved in cisplatin chemoresistance via Wip1, a suppressor of the ATM-dependent signaling pathway. Wip1 silencing attenuated DNA damage repair and strengthened the cisplatin chemosensitivity of oral squamous cell carcinoma cells [69]. Several chemoresistance signatures of breast carcinoma cells were closely related to the strand separation and nuclease activities of YB-1 [70]. Protein reversionless 3-like (REV3L) was found to function as a catalytic subunit of DNA polymerase (pol) ζ, and take responsibility for error-prone translesion synthesis, so its low expression enhanced the chemosensitivity of esophageal squamous carcinoma cells to 5-FU, evidenced by G_1_ phase arrest and apoptotic induction [71].

### Autophagy

**Figure 2:**
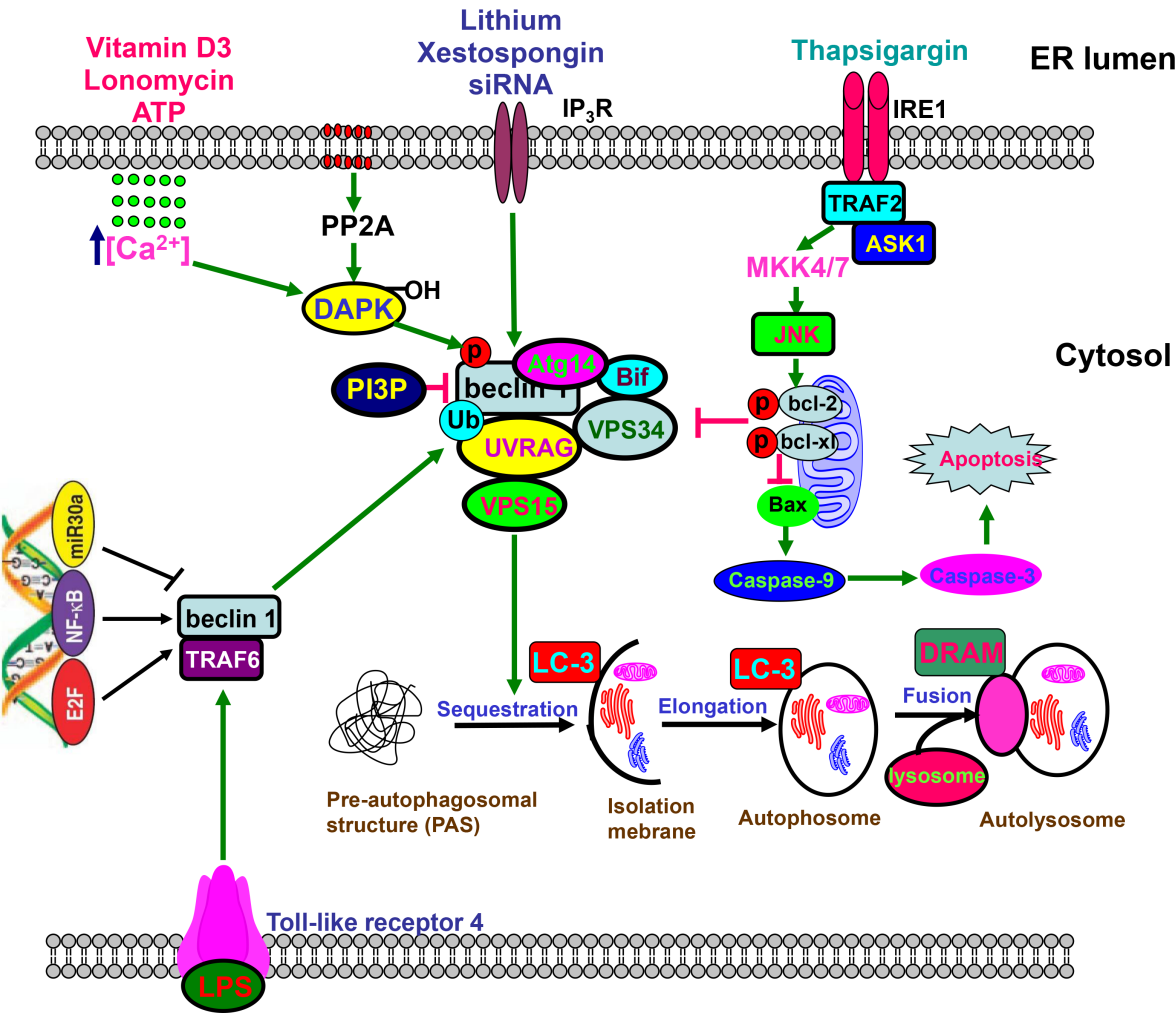
Beclin 1-centered autophagy Beclin 1 functions as a scaffold for the formation of autophagosome via interaction with Atg14, URAG, Bif, Vps34, and VPS15, which can be disrupted by phosphorylation of Bcl-2 and Beclin 1, or ubiquitination of Beclin 1. The core complex, which is negatively modulated by PI3P, undergoes sequastration and enlongation to form autophosome containing organells and macromolecules, finally to produce autolysome via the fusion with lysosome. Toll-like receptor 4 ubiquitinates Beclin 1 at Lys63 via TNF receptor associated factor 6. Either JNK1-mediated Bcl-2 phosphorylation or DAPK-mediated Beclin 1 phosphorylation causes the dissociation between Beclin 1 and Bcl-2 for the reversal of Bcl-2-mediated autophagic inhibition. Beclin 1-mediated macroautophagy involves the regulation of Caspase-9 expression. However, Caspase 8- or Caspase 3-mediated cleavage of Beclin 1 reduces autophagy and promotes apoptosis. E2F and NF-κB could bind to the promoter of Becn1 for its up-regulation, while versa for miR30a.

After cells suffered from nutrient starvation, hypoxia, LPS and chemotherapy, autophagy is initiated to degrade cellular damaged organelles or particles, and recycle amino acids or fatty acids via autophagosome formation (Figure 2). There are four pathways of autophagy, including macroautophagy, microautophagy, chaperone-mediated autophagy, and mitophagy. Cellular stress or increased metabolic demand activates autophagy, whose adaption can promote cell survival, and cause tumor growth and therapeutic resistance. Therefore, autophagic inhibition restores chemosensitivity and increases cancer cell death using hydroxylchloroquine (HCQ) or its derives [72].

Beclin 1 was found to be highly expressed in neuroblastoma, and a combination of HCQ and vincristine significantly decreased its progression. As a matter of fact, chemoresistance was positively linked to enhanced protective autophagy and weakened apoptosis in bladder cancer cells, treated with Gossypol [73] and osteosarcoma cells treated with cisplatin [74]. Amantini et al. [75] found that Capsaicin triggered autophagic cell survival, and drove EMT and chemoresistance in bladder cancer cells in Hedgehog-dependent manner. NFKB1/NF-κB pathway was activated by cistplain to induce GFRA1 expression for AMPK-dependent autophagy [74]. In addition, doxorubicin, cisplatin and etoposide induced the expression and cytosolic translocation of HMGB1 to elevate autophagy level of neuroblastoma cells. HMGB1 overexpression also contributed to the chemoresistance of neuroblastoma cells by inducing Beclin-1-mediated autophagy [76]. Yang et al. [77] found that HMGB1 enhanced starvation-dependent autophagy of leukemia cells through PI3K/Akt/mTORC1 pathway, and 3-MA reduced the autophagy and chemoresistance of leukemia cells.

Reportedly, autophagy was demonstrated to make HCC cells resistant to chemotherapy under hypoxia, which was inhibited by 3-MA or Beclin 1 siRNA [78]. AQP3 was involved in cisplatin resistance via LC3-I-to-II conversion, Atg5 and Beclin 1 overexpression in gastric cancer cells [79], and heparanase within autophagosomes contributed to autophagy and resistance chemotherapy [80], both of which were reversed by CQ. Piya et al. [81] found that Atg7 silencing in acute myeloid leukemia enhanced the sensitivity to the genotoxic agents with alteration of Bcl-2 family proteins and up-regulation of PMAIP1/NOXA mRNA. CD44v6 overexpression mediated the acquired chemoresistance to 5-FU and oxaliplatin by inducing autophagy and EMT, and activating both PI3K-Akt and Ras-Erk signal pathways in colon cancer cells [82]. Xu et al. [83] showed that miRNA-30a knockdown activated Beclin 1- mediated autophagy for osteosarcoma chemoresistance. miR-200b down-regulated Atg12 expression, which suppressed the autophagy and docetaxel resistance of lung cancer cells [84]. Src/Stat3- induced HO-1 overexpression mediated the doxorubicin resistance of breast cancer cells via autophagic induction [85]. Active EGFR also bound to and phosphorylated Beclin 1 for the latter's inhibitor binding and decreased Beclin 1-associated VPS34 kinase activity for chemoresistance to TKI therapy [86].

### EMT

**Figure 3:**
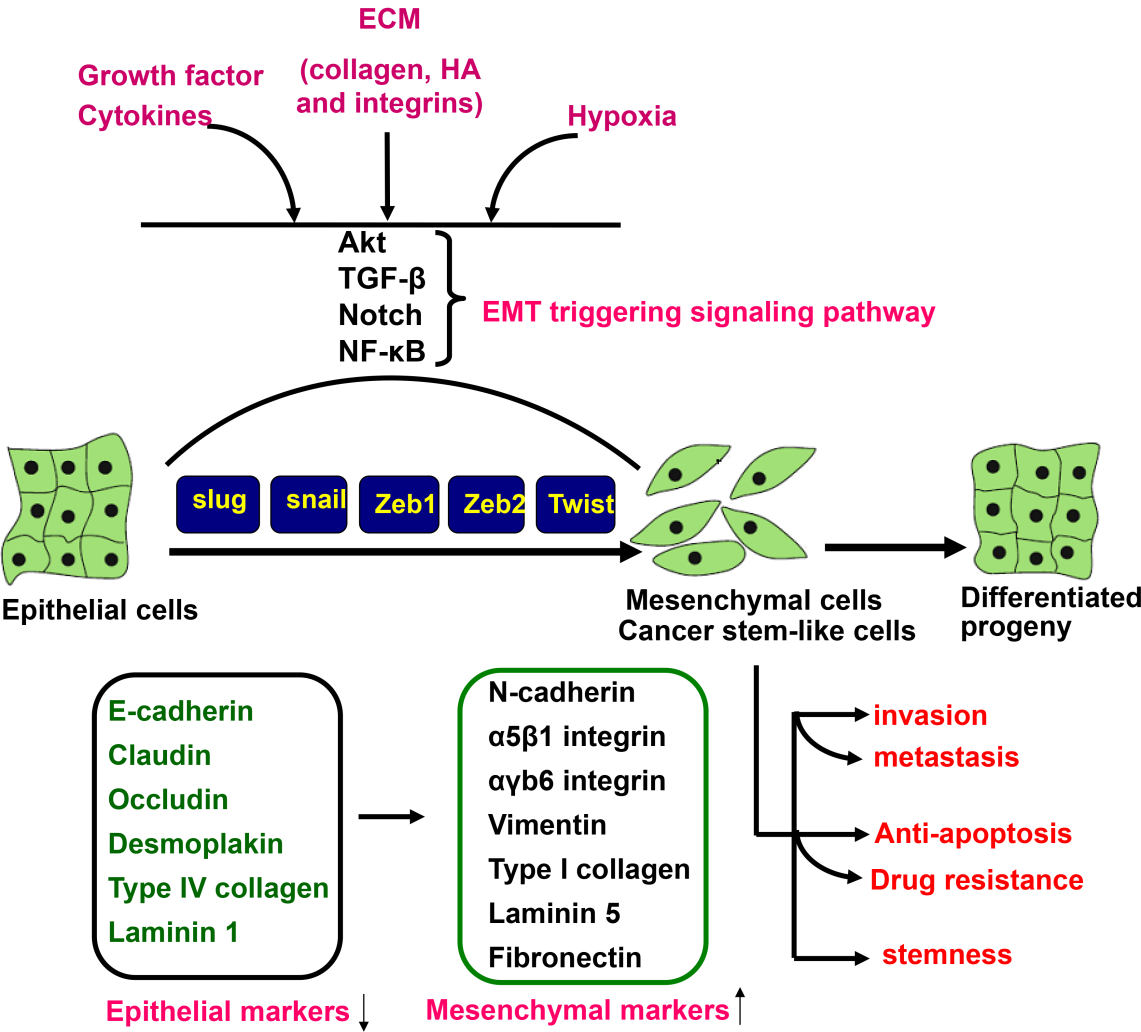
EMT triggering and signal transduction EMT is triggered by the interplay of extracellular signals (collagen, hyaluronic acid and integrin), growth factors and cytokines (TGF-β, VEGF, EGF and HGF), and hypoxia. The receptor- mediated signal pathways involve Akt, smad, Notch and NF-κB, finally to up-regulate a set of transcription factors including snai1, slug, Zeb1, Zeb2, and Twist, which regulate the expression of epithelial and mesenchymal markers at a transcriptional level. Consequently, there appear the down-regulation of epithelial markers (E-cadherin, claudin, occludin, desmoplakin, type IV collagen, and laminin 1) and up-regulation of mesenchymal markers (N-cadherin, intregrin, vimentin, type I collagen, laminin 5, and fibronectin). The mesenchylmal stem -like cells displayed the aggressive phenotypes, including invasion, metastasis, anti-apoptosis, drug resistance and stemness. HA, hyaluronic acid; ECM, extracellular matrix.

As shown in Figure 3, EMT is a process by which epithelial cells lose cell polarity and homogenous adhesion, and gain migratory and invasive properties to become mesenchymal stem cell. It was triggered by cytokines and growth factors, non-coding RNAs or hypoxia and characterized by dissociation of cell–cell contacts, alteration of the cytoskeletal network, and increased proteolytic activity, consequently leading to cell invasiveness, anchorage-independent growth (anoikis), apoptotic and drug resistance in several types of cancer [87].

**Figure 4:**
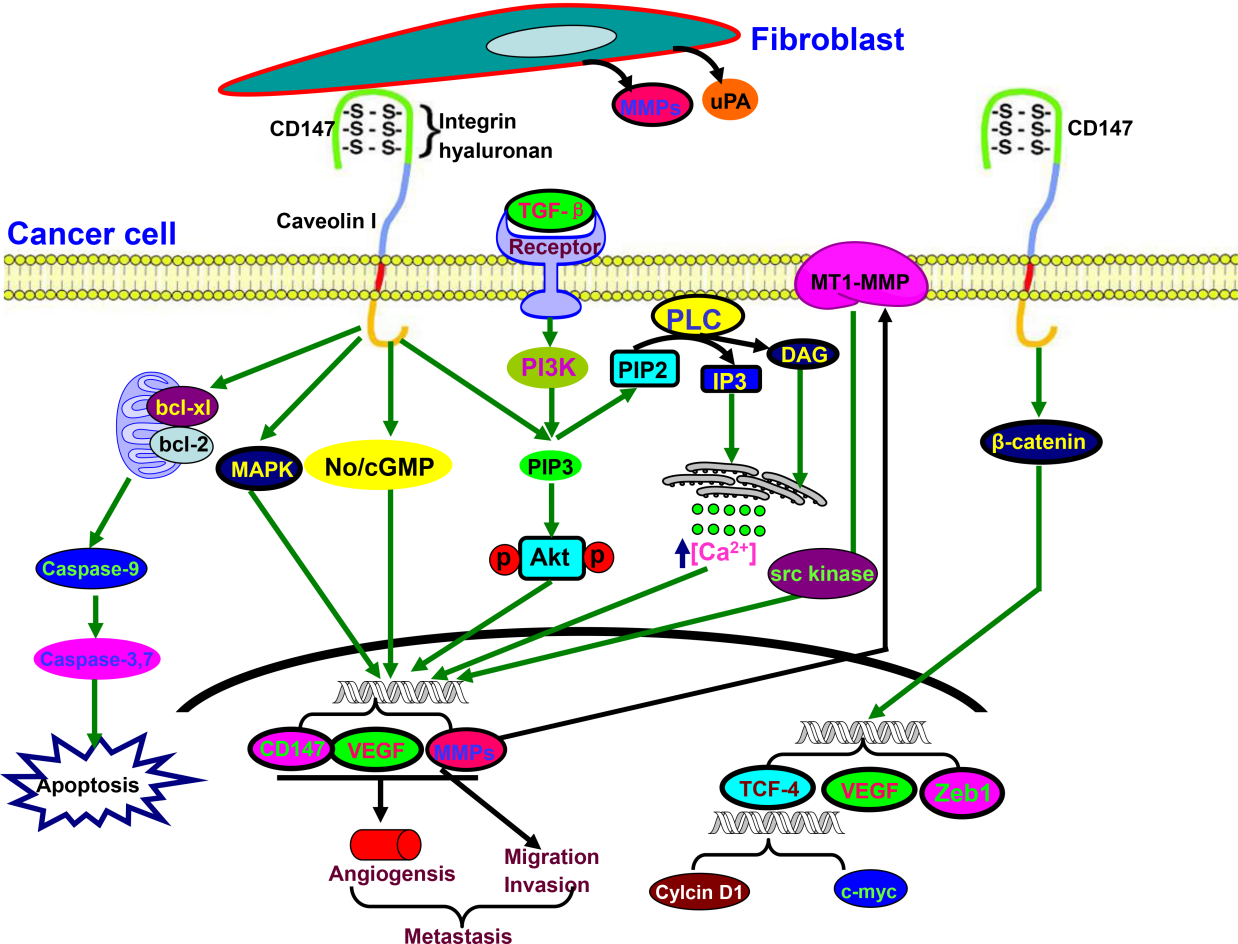
The biological functions of CD147 Activated CD147 might increase tumor invasion by inducing the release of MMPs and uPA in the surrounding stromal cells. It also stimulates tumor angiogenesis by elevating VEGF and MMP expression in neighboring fibroblasts and epithelial cells *via* microvesicle shedding. Hyaluronan, caveolin, or integrin α3β1 interacts with CD147 in cancer cells and enhances the effect of CD147 on adhesion, invasion and MMPs secretion. Up-regulated CD147 expression is induced by TGF-β coupled with epithelial-mesenchymal transition, and targets PIP3, No/cGMP, and MAPK to induce EMT for cancer invasion and metastasis. Additionally, CD147 suppresses the mitochondria-dependent apoptosis via Bcl-2 and Bcl-xL. CD147 expression is controlled by the cell survival PI3K/Akt/GSK3β signaling pathway, and is directly regulated by the transcription factor Slug. CD147 overexpression promotes the nuclear translocation of β-catenin, which up-regulates the transcriptional level of TCF-4, VEGF and Zeb1.

CD147 is a glycosylated transmembrane protein and also known as extracellular matrix metalloproteinase inducer (Figure 4). It can interact multivalently with CD44 and cause assembly or stabilization of signaling and transporter complexes within specialized lipid raft, containing MT1-MMP, ErbB2, EGFR, ABC-family transporters and MCTs responsible for anti-apoptotic signaling, cell proliferation, invasiveness, and chemoresistance [88]. In HCC, CD147 overexpression initiated a TGF-β signaling cascade, including Slug expression, cadherin switching, and morphological EMT changes [89]. Xiong et al. [90] confirmed that CD147 suppression enhanced the effects of trastuzumab of HCC cells by up-regulating the cleavage of Caspase-3/9 and PARP, and down-regulating MAPK and Akt phosphorylation.

Nanog overexpression directly resulted in EMT of ovarian cancer cells with a low susceptibility to cisplatin by activating Stat3 pathway and up-regulating the expression of MDR-1 and GST-π [91, 92]. Zhang et al. [93] found that nestin overexpression was positively associated with EMT and chemoresistance of HCC with the activation of Wnt/β-catenin signaling.

Lee et al. [94] demonstrated that NNK increased chemoresistance in colorectal cancer via up-regulation of Snail, CD133, Nanog, Oct4, and drug-resistant genes. Doxorubicin chemoresistance induced EMT via up-regulation of TGF-β signaling in colon cancer cells [95]. It was the same for gemcitabine chemoresistance in hepatoma cells via PDGF-D pathway [96]. Pantoprazole, a proton pump inhibitor, inhibited growth viability, self renewal and 5-FU resistance of cancer stem cells from gastric cancer cells via EMT/β-catenin pathways [97].

Hu et al. [98] found that miR-760 mediated chemosensitivity of breast cancer to doxorubicin through inhibition of EMT. miR-138, miR-145 and miR-489 suppressed chemoresistance and EMT in lung, hepatocellular and breast cancers by targeting Zeb2 and Smad3 respectively [99–101]. miR-203 down-regulation was responsible for chemoresistance in glioblastoma by promoting EMT via Snail2 [102]. Li et al. [103] showed that LncRNA SLC25A25-AS1 knockdown remarkably promoted the drug resistance and EMT of colorectal cancer cells through Erk and p38 activation.

### Cancer stem cells

Cancer stem cells (CSCs) are a cell subpopulation of cancer cells, and characterized by tumor forming capacity, self-renewal, multiple differentiation, drug resistance, cancer growth and recurrence. These cells are believed to cause relapse and metastasis by giving rise to new tumors. Generally, flow cytometry and PI staining were employed to sort a side population (SP) in leukemia, breast cancer, lung cancer and glioma as stem-like cells [87]. CSCs were characterized to efflux Hoechst 33342 or PI via multidrug resistance (MDR) and ATP-binding cassette transporters. Therefore, CSCs have been found to resist chemotherapy due to the overexpression of P-gp, ABCG2, Bcl-2 and survivin.

The reciprocal correlation between CSCs and chemoresistance, evidenced weakened chemosensisivity and strengthened stemness. Kulsum et al. [104] found that head and neck squamous cell carcinoma cells resistant to cisplatin, 5-FU or docetaxel showed the hyperexpression of stem cell marker proteins (CD44, CD133, ALDH1A1, NOTCH1, Oct4 and SOX2) and displayed an increase in migration and invasion, spheroid, colony and tumorigenic formation, which was reversed by ALDH1A1 knockdown. Reportedly, CD133^+^ colorectal cancer cells had chemoresistance to 5-FU via survivin and ABCG2 expression [105], and ABCG2 knockdown inhibited the self-renewal capacity of these cells, and enhanced chemotherapy-induced apoptosis [106]. Izumiya et al. [107] found that 5-FU-pretreated cells were capable of initiating spheres and metastasizing, and overexpressed stem cell marker genes, Oct4 and Nanog. It was noted that morphine enhanced the mammosphere forming capacity, SP cell enrichment, chemoresistance and the expression of stemness-related transcription factors (Oct4, Sox2 and Nanog) of cancer cells via Wnt/β-catenin activation [108], indicating that we should be careful to employ morphine for cancer pain relief. However, genistein treatment was demonstrated to reduce the chemoresistance of gastric cancer cells to 5-FU and cisplatin via inhibition of ABCG2 expression and Erk 1/2 activity [109].

Reportedly, hyaluronan (HA) increased the expression of histone methyltransferase DOT1L in CSCs of head and neck cancer, which were characterized by CD44v3 and ALDH1 hyperexpression. DOT1L knockdown blocked the expression of DOT1L, miR-10b, RhoGTPase and survival protein, and reduced cancer invasion and chemoresistance in CSCs [110]. Govaere et al. [111] identified laminin-332 as part of the specialized CSC niche of HCC cells to maintain chemoresistance and quiescence. Jung et al. [112] reported that stem cell reprogramming factor, PBX1, was positively correlated with shorter post-chemotherapy survival in ovarian cancer patients. PBX1 overexpression promoted cancer stem cell-like phenotypes by binding to stat-3 for transcriptional up-regulation. Lgr5 overexpression was reported to associate with poor response to 5-FU-based treatment in colorectal cancer, and increased stem cell property and chemoresistance to 5-FU and oxalipatin by up-regulating ABCB1 expression [113]. In Sp cells, there appeared up-regulated expression of miR-21 and its upstream regulator AP-1 transcription factors, whose inhibitor or antagonist increased chemosensitivity and decreased colony forming ability [114].

### Exosomes

Exosomes are cell-derived 30-100nm microvesicles filled with blood, urine, and culture medium, even macromolecules including DNA fragments, mRNAs, proteins and miRNAs. They are either released from the cell when multivesicular bodies fuse with the plasma membrane or released directly from the plasma membrane, finally to mediate cell-cell communication. The exosome contents from cancer cells, cancer-associated fibroblast (CAF) or mesenchymal stem cells may be shuttled from donor to recipient cells, and control a wide range of pathways, such as tumor growth, development, metastasis, angiogenesis, drug resistance and premetastatic niche preparation by establishing a fertile microenvironment, suppressing immune surveillance and removing chemotherapeutic drugs. Therefore, exosomes might be potential targets for therapeutic interventions and potential markers for therapeutic and prognostic prediction of cancer [115, 116].

Reportedly, exosomes derived from breast cancer cells could enhance active sequestration of drugs and mediate drug resistance by transferring MDR-1 and P-gp protein, which was inhibited by Psoralen [116]. Kreger et al. [117] discovered that paclitaxel exposure caused breast cancer cells to generate survivin-containing exosomes, which strongly lengthened the survival of serum-starved and paclitaxel-treated fibroblasts and breast cancer cells. Receptor potential channel, TRPC5, mediated chemoresistance and was transferred to chemosensitive breast cancer cells through releasing TRPC5-containing exosome [118]. Chen et al. [119] also found that the acquired chemoresistance was due to intercellular transfer of specific miRNAs by releasing exosomes. Li et al. [120] proposed that exosomal miRNAs regulated the translation of AR, PTEN and TCF4 genes in chemoresistant prostate cancer cells.

MSC exosomes were reported to induce 5-FU resistance of gastric cancer cells to by antagonizing 5-FU-induced apoptosis, activating CaM-Ks/Raf/MEK/Erk pathway, and promoting MDR, MRP and LRP expression [121]. Richards et al. [122] found that CAFs were intrinsically resistant to gemcitabine, and released exosomes by gemcitabine exposure, which promoted the proliferation and drug resistance of recipient epithelial cells by up-regulating snail. Hu et al. [123] found that fibroblast-derived exosomes promoted proliferative percentage, clonogenicity and tumor growth of CSCs upon treatment with 5-FU or oxaliplatin. However, cisplatin uptake by cancer cells and exosomal cisplatin content was markedly impaired by low pH conditions or PPI (proton pump inhibitor) treatments [124]. Additionally, Zhang et al. [125] found that β-elemene- induced exosomes reversed drug resistance of breast cancer by increasing PTEN expression and decreasing Pg-p expression in cells and exosomes.

## CONCLUSIONS AND PERSPECTIVE

The review may provide further understanding of chemoresistance signal network, which facilitates the establishment of valid therapeutic targets and potential chemosensitivity biomarkers in cancer therapeutics. To roll back the cancer recurrence and increase the patient lifespan, we summarized the past and future of chemoresistance as follows:

1. Chemoresistance-related proteins are localized to extracellular ligand, membrane receptor, cytosolic signal messenger, and nuclear transcription factors.

2. These proteins are involved in various events, including proliferation, apoptosis, EMT, autophagy and exosome.

3. There is cross-talk between these proteins: EGFR-Akt-NF-kB-genes (Bcl-2, Bcl-xL and suvivin) or EMT-related stemness.

4. It is possible and essential for the cancer patients to receive target, individualized and combine therapy and to increase therapeutic efficacy and decrease tissue toxicity.

5. Due to tissue- or cell- specificity of chemoresistance, it is important to build up the screening system of the biomarker of chemosensitivity and chemoresistance using cancer patient samples by immunohistochemistry, RT-PCR, cDNA microarray or transcriptomic sequencing.

6. *In vitro* cell cultures and *in vivo* orthotopic tumors should be emphasized to clarify the contribution of microenvironment to drug resistance, and might be employed to screen the efficacy of chemoreagents.
